# Self-Management of Geriatric Syndromes–longitudinal data on medical and psychosocial factors in older patients

**DOI:** 10.1038/s41597-026-07405-x

**Published:** 2026-05-28

**Authors:** Aline Schönenberg, Konstantin G. Heimrich, Rebecca Wientzek, Natalie Berges, Annika Sternkopf, Antonia Schindler, Tino Prell

**Affiliations:** 1 Department of Geriatrics, Jena University Hospital, Jena, Germany; 2 Department of Geriatrics, Halle University Hospital, Halle, Saale Germany

**Keywords:** Outcomes research, Geriatrics

## Abstract

Older patients who are acutely ill are a vulnerable group who are often excluded from scientific research. Nevertheless, psychosocial and health-related changes pose a particular challenge for this group that must be understood as our society continues to age. Geriatric syndromes, which are disease-unspecific, may particularly affect disease management and well-being. This dataset contains longitudinal data on 666 geriatric patients recruited from geriatric wards and outpatient practices. At baseline, routine data containing geriatric assessments (evaluating cognition, mood, mobility, daily activities, nutrition) and medical information were collected, as well as data from questionnaires regarding demographics, geriatric syndromes, self-management, loneliness, quality of life (WHOQOL-Bref), self-efficacy and attitudes towards ageing. During telephone follow-ups after three and six months, survival was recorded, as well as changes in health, geriatric syndromes and healthcare usage. Measures of quality of life, self-management and views on ageing were repeated. The dataset can be used for a variety of analyses investigating the personal, social and institutional factors that influence health and well-being in older patients.

## Background and Summary

According to the World Health Organization (WHO), health is no longer defined solely as physical health but also includes social connectedness and mental well-being^1^. This approach is mirrored in the biopsychosocial model of illness, describing health to be an individual interaction of biological procedures, social parameters and psychological well-being^2^. Especially in advancing age, where inevitable biological decline may tamper with physical health, an extended view of health is beneficial to ensure continuously high quality of life (QoL)^3–5^.

Older adults constitute a particularly vulnerable group, as advancing age brings with it a unique combination of growth and decline^6^; while time and financial resources as well as resilience, coping strategies and positive views of ageing^7,8^ may compensate for decreasing health and uphold well-being for many years, physical and cognitive decline is inevitable due to increasing biological vulnerability in older age^9,10^. This physical decline is often accompanied by reduced functionality and autonomy, leading to social isolation^11^ and mental health problems^11,12^. In addition, older, acutely ill patients are not only challenged with the respective acute illness or worsening thereof, but often carry with them a multitude of diagnoses, leading to multimorbidity^13–16^. This is why the field of geriatric medicine has shifted away from individual diseases towards a comprehensive focus on overall geriatric syndromes^9,15,17^. According to Inouye, *et al*.^17^, geriatric syndromes can be interpreted as cumulative effects of multiple impairments, encompassing a range of issues including mobility difficulties, cognitive decline, incontinence, and others that collectively increase vulnerability and impact the QoL of older patients^14,18,19^.

Thus, older adults are faced with the challenge of managing multiple, interrelated health concerns while potentially dealing with cognitive decline, culminating in reduced self-efficacy and self-management^20–22^. Self-management of health, however, is an important cornerstone for QoL in advancing age, especially in the face of a healthcare system that is not yet equipped to handle the expected increase in older patients^23^. Good self-management may act preventively and improve treatment outcomes by allowing patients to monitor their health and symptoms and act appropriately^24–27^. Still, older adults are faced with multiple challenges that may impact their self-management such as multimorbidity, cognitive decline, and lack of social support^22,28^, making self-management a tool that must be understood in depth in order to provide tailored support.

Consequently, a deeper understanding of multi-layer mechanisms influencing health and well-being in older age is especially relevant in the face of a current demographic shift towards an ageing society. The WHO estimates that by 2050, around 2 billion people worldwide will be aged 60 or above^9^, yet in contrast to this increasing number of older adults, the healthcare system is not yet adapted to their care^2,29,30^. In Germany, a 2016 review of medical guidelines issued by the German Association of Scientific Medical Societies revealed that only a handful of more than 900 guidelines directly addresses the health of older adults^30^, partially because older adults are systematically excluded from clinical studies^31^. Much research performed on health and well-being of older adults is often focused on healthy, independent community-dwelling older adults, however, data on hospitalized, institutionalized or severely ill older persons is rare^31^.

In the presented dataset, we therefore combine data from three geriatric hospital wards and two general practitioner (GP) practices to provide longitudinal information on health and well-being of older acutely ill adults. During the study, N = 666 geriatric patients were assessed with a comprehensive geriatric routine assessment and additionally responded to questionnaires on physical and mental health, QoL, social connectedness, medication adherence, self-management, and self-efficacy. Follow-up data provides further information on the development of these patients after 3 and 6 months. With its multicenter and longitudinal approach, this study provides encompassing data on a systematically excluded patient population^31^, serving as a potential base for a variety of research questions in this vulnerable cohort. The unique combination of clinical data and questionnaires invites a plethora of research questions for a holistic understanding of health in older age: from views of aging and its relationship with QoL and mortality, over self-efficacy and its effect on health behaviour and outcomes, to loneliness and its association with cognition or depressiveness. The data additionally provides descriptive information on health status such as functional health and cognition, care level, and modes of care (nursing services, nursing homes, use of aids, use of nonmedical treatment etc), making it an ideal dataset for comparison with researchers’ own data.

## Methods

This dataset presents observational, longitudinal data on geriatric patients with the aim to characterize self-management of geriatric syndromes and related psychosocial and health-related factors.

Data were collected as part of the SelfManGer study, which examined the self-management of geriatric patients in Germany^24^, and was conducted at geriatric wards in two hospitals in Saxony-Anhalt (German clinical trial registration: DRKS00031016). The related JenaGer study examined the self-management of geriatric patients in Thuringia (German clinical trial registration: DRKS00032328).

In addition, SelfManGer recruited patients from two collaborating GP practices in urban areas. Although the SelfManGer and JenaGer studies had different overall aims, they were designed with overlapping instruments to ensure two comparable core datasets. Baseline data collection took place between February 2023 and August 2024, with telephone follow-up taking place after three and six months (SelfManGer only). It should be noted that, although the SelfManGer and JenaGer studies both contain more data than is presented here, a common set of comparable items and questionnaires was defined to enable the studies to be merged into a single dataset. This manuscript provides data on comparable study components.

All patients were subjected to a preliminary screening process, which was based on a review of their existing medical records. Those patients who met the eligibility criteria were then approached by members of the study team, who provided them with detailed information about the study. The study was approved by the local ethics committee of Halle University Hospital as well as the State Chamber of Physicians of Saxony-Anhalt (Landesärztekammer; approval number: 2022–026) for SelfManGer, and the ethics committee of Jena University Hospital (2023-2923-BO) for JenaGer. In instances where patients had provided written informed consent, study staff proceeded to administer the questionnaires in conjunction with each patient. The study incorporated both questionnaires and medical information obtained from geriatric assessments conducted by trained hospital staff as part of routine inpatient care.

### Setting and participants

For inpatients, hospitalized older adults were recruited from the geriatric wards of three hospitals in Saxony-Anhalt and Thuringia, Germany. All patients were treated with specialized geriatric care as defined by the German procedure classification (Operationen- und Prozedurenschlüssel - OPS), the official classification for the encoding of operations, procedures and general medical measures, and were thus classified as “geriatric” patients by geriatric medical specialists. This early geriatric rehabilitation treatment spans over two weeks on average and is performed by a multidisciplinary team including geriatric specialists, nurses, physiotherapists, occupational therapists, speech therapists, social workers, and psychologists. Patients from the two GP practices were classified as “geriatric” based on age, multimorbidity and the medical expertise of the recruiters.

Baseline recruitment took place between February 2023 and August 2024; with follow-up after 3 months (FU3) taking place between May 2023 and November 2024 and follow-up after 6 months (FU6) from August 2023 to February 2025.

At baseline, we combined study-specific questionnaires with routine data collected during hospital stay. All patients routinely received a comprehensive geriatric assessment performed by medical staff as part of hospital care; this includes assessments of cognition, depressive mood, mobility, daily activities and functional status, nutrition, and handgrip strength depending on the available routine data for each recruitment centre (Table 1). In addition, we extracted medical information such as height and weight, diagnoses, and medication from medical records. The study-specific questionnaires contain information on geriatric syndromes (patient-reported), self-management, self-efficacy, social connectedness, QoL, views of ageing, and medication adherence (Table 1). In addition, we assessed sociodemographic data and perception of geriatric syndromes using self-constructed questions (see Supplement).

**Table 1:** Included measures and questionnaires.

Geriatric Assessment		
Instrument and Source	Description	Location
Functional status: Barthel Index^48^	10 items depicting the ability to perform daily tasks such as washing, walking and dressing, measured upon admission and discharge	All hospitals
Geriatric Depression Scale (GDS)^49,57^	15 dichotomous items depicting depressiveness in older adults	All
Geriatric Screening according to Lachs^51^	16 dichotomous items depicting pathology in different areas such as cognition, mobility, hearing and vision	All hospitals
Handgrip Strength	Measurement of hand grip using JAMAR dynamometer in KG	Hospital 1,3
Nutritional Status Nutritional Risk Screening (NRS)^58^ Mini Nutritional Assessment Short Form (MNA-SF)^59^	Classifying patients into at risk of malnutrition vs not at risk	All hospitals
Mini Mental State Examination (MMSE)^52^	Cognitive screening tool	Hospital 2, 3
Mini Cognitive Status (MiniCog)^40^	3-part cognitive screening on recall and clock test	Outpatients
Montreal Cognitive Assessment (MOCA)^60^	Cognitive screening tool. MOCA scores were transformed into their equivalent MMST scores^39^	Hospital 1
Tinetti Test^42^	2-part test to rate mobility and stability performed at admission and discharge	All hospitals
Timed Up and Go Test^41^	Walking speed test performed at admission and discharge	All hospitals
Weight, Height, BMI		All
Diagnosis	Main diagnosis upon admission	All
Number of Medications	HOSP2: admission, other: discharge	All
**Study-specific questionnaires**		
Appraisal for Self-Care Agency Scale (ASAS)^34,46^	15 items enquiring after steps that have been taken to self-manage health	All
Beck Anxiety Index (BAI) short^47^	5 items enquiring after physical and cognitive anxiety symptoms as taken from the SHARE survey^61^	All hospitals
Beliefs about Medication Questionnaire (BMQ)^62^	Containing 2 items on medication adherence, 2 items on medication beliefs and 2 items on concerns	All hospitals
Generalized Self-Efficacy Scale (GSE)^50^	10 items on self-efficacy	All hospitals
Quality of Life (WHOQOL-Bref)^44,45^	26 items on 4 areas of QoL: physical, mental, social and environmental	All
UCLA-Loneliness Scale- Short Form^53,63^	3-item version depicting lack of companionship, feeling isolated and feeling left out	All
Views on Ageing^54,64^	General subjective aging perception, 5 items taken from the German Ageing Survey in accordance with the Philadelphia Geriatric Center Moral Scale^65^	All
**Geriatric Syndromes**		
Presence of geriatric syndromes: yes/No	Mobility problems, falls, cognition problems, loneliness, depressiveness, pain, sleep problems, incontinence, dysphagia (problems with swallowing)	All
Which of these syndromes is the most restrictive in your daily life?	Mobility problems, falls, cognition problems, loneliness, depressiveness, pain, sleep problems, incontinence, dysphagia (problems with swallowing	
How restricted are you due to this syndrome?	0–100	
How confident are you that the syndrome will improve? How much do you expect it to worsen?	0–100	
Would you attribute the syndrome to age or illness?	0–100	
Use of electronics	Yes/No for Smartphone, Tablet, Computer/Laptop, Telephone	Not HOSP2

Follow-up was performed via telephone, as the majority of patients were unable to return to the clinic for health-related reasons. Survival was the main variable of interest. In addition, we recorded rehospitalization, changes in health, healthcare usage, geriatric syndromes, depressive mood, functional status, self-management, VOA, and adherence (see Supplement).

Furthermore, a questionnaire was constructed in order to gather data on healthcare usage, geriatric syndromes and sociodemographic information (please refer to the supplementary materials for a translation of the full questionnaire). In this questionnaire, patients were invited to indicate the presence (yes/no) of nine different geriatric syndromes, followed by the question of which of these syndromes they considered to be the most restrictive in terms of their daily lives. In relation to the aforementioned syndromes, patients were invited to indicate their perceived level of restriction (0–100), their expectations regarding the improvement or deterioration of the syndrome (0–100), and whether they attributed the syndrome to age or illness (0–100).

The collection of data was overseen by a central team of trained study staff at all locations, with the objective of ensuring comparable data quality irrespective of the location from which the participants were recruited. All patients were initially screened for study eligibility. The inclusion criteria encompassed treatment in geriatric care, age ≥70 or 65 with multimorbidity (*SelfManGer*), and the capacity to perform basic self-management activities. Patients were included in the study if study and hospital staff deemed them capable of holding a meaningful conversation and comprehending the questionnaires^31^. The exclusion criteria encompassed cases where subjects were unable to perform any self-management, such as those who were completely bedridden and dependent on ADLs, as well as those with acute delirium or severe dementia, as diagnosed by medical professionals. Upon admission, each patient was subjected to a screening process, the results of which were derived from their medical records. Patients who met the necessary criteria were then approached by members of the study staff, who informed them of the study procedure. Once more, cognitive eligibility was evaluated on the basis of this conversation and subsequent consultations with nurses. For outpatients, similar criteria were applied: patients aged aged ≥70 or 65 with multimorbidity and at least one geriatric syndrome, but without delirium or severe dementia with at least one geriatric syndrome were randomly selected. The exclusion criteria of inability to perform self-management also remained, however, this was less relevant in outpatients. In the event that patients were deemed eligible for participation in the study and provided written informed consent, the study staff proceeded to administer the questionnaires to each patient. This process entailed the staff reading the questions aloud and documenting the patients’ responses. It was imperative for the majority of patients to undergo this procedure due to their impaired visual acuity, concentration, and motor skills. These impairments significantly hindered their capacity to comprehend and complete extensive questionnaires^31^.

**Fig. 1:**
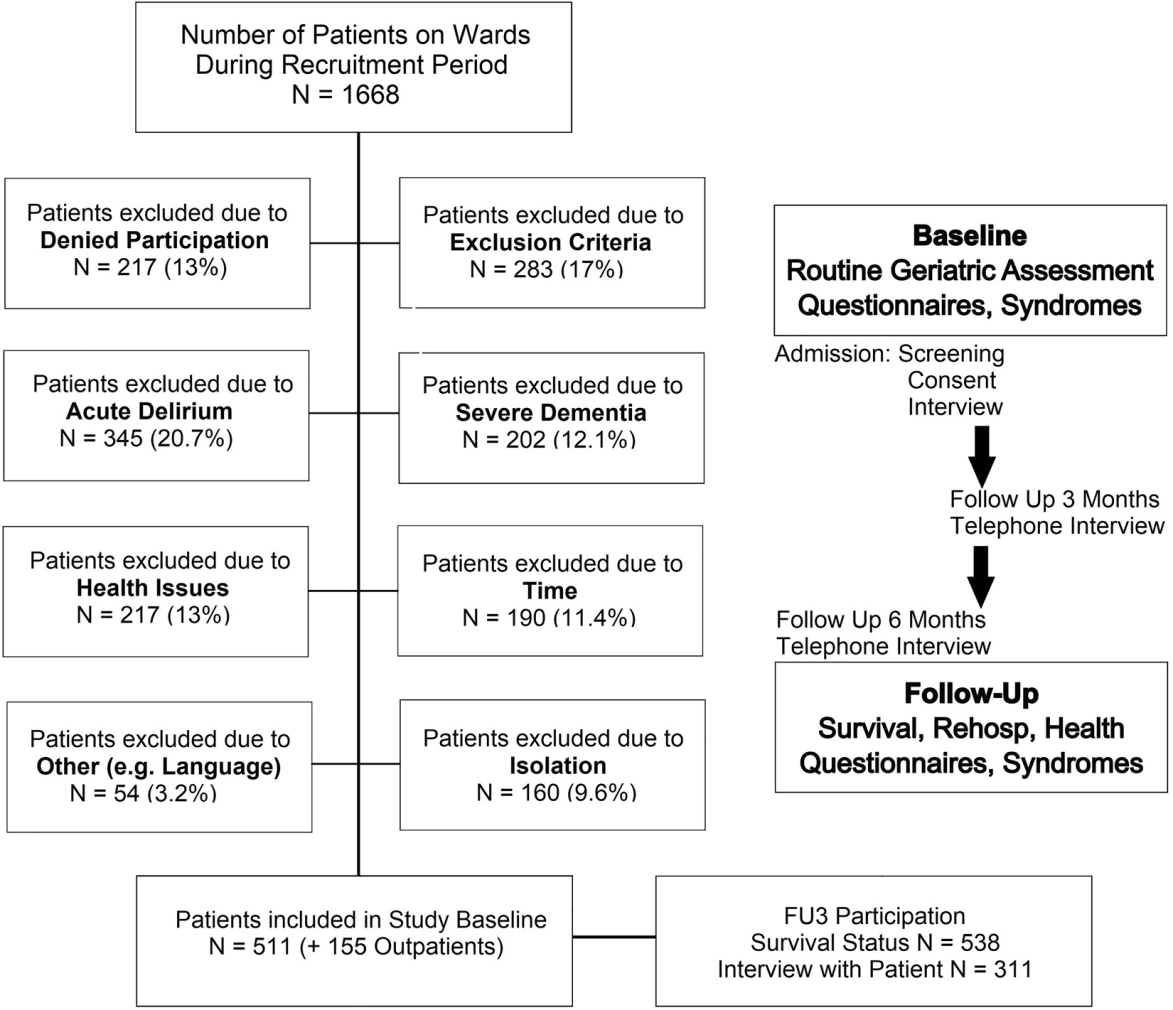
Flow chart of recruitment and study procedure.

During the recruitment period, a total of N = 1668 patients were admitted to the geriatric wards (Fig. 1). Around half of these patients were deemed unfit for participation based on exclusion criteria (N = 283 due to inability to perform self-management skills, N = 202 due to severe dementia, N = 345 due to acute delirium). Although potentially eligible, N = 190 patients were missed due to timely reasons (discharged in advance, at examinations, had visitors) and N = 160 patients were unavailable for participation as they were in isolation due to infections. Furthermore, N = 217 patients were unable to complete the assessment due to other medical reasons such as vigilance disorders, severe hearing problems, speech disorders, or mortality on the ward, and N = 54 had other reasons such as language barriers impeding their participation. Lastly, N = 217 patients did not provide consent to participate. Since these patients did not consent to having their data collected, no comparison between patients who declined and patients who participated is possible in retrospect, potentially introducing a bias in the dataset.

Of the 666 included patients aged 82.1 (SD 6.3) years, the majority were female (64.3%) and widowed (54.5%). Patients had between 2 and 52 diagnoses with an average of 15.6 (SD 7.7), and 3.4 geriatric syndromes (SD 1.8). Most patients had medium to high education levels and showed mild cognitive deficits according to the MMST. Table 2 presents an overview of the included patient cohort.

**Table 2:** Brief Cohort Description.

Variable	Mean (SD)	Median (IRQ)	Range
Age	82.1 (6.29)	83 (8)	61–96
MMSE	25.2 (3.31)	26 (5)	10–30
GDS	4.16 (3.11)	4 (4)	0–15
Barthel Admission	46.3 (18.8)	45 (25)	5–100
Barthel Discharge	69.3 (19.2)	75 (30)	10–100
Number of Geriatric Syndromes	3.41 (1.84)	3 (3)	0–9
Number of Diagnoses	15.6 (7.72)	15 (10)	2–52
**Variable**	**Level**	**Count**	**Percentage**
Location	HOSP1	155	23.3
	HOSP2	232	34.8
	HOSP3	124	18.6
	GP	155	23.3
Gender	Male	238	35.7
	Female	428	64.3
Education	Low (<10 years)	17	2.6
	Middle (<12 years)	354	53.2
	High (≥12 years)	291	43.7
Living	Alone	326	48.9
	Partner/Family	280	42.0
	Other	58	8.7
Marital	Single	44	6.6
	Married/Partner	256	38.4
	Divorced/Widowed	363	54.5
Carelevel	0 (none)	294	44.1
	1	66	9.9
	2	173	26.0
	3	104	15.6
	4	11	1.7
	5	2	0.3
Satisfaction with health	1 very dissatisfied	73	11.0
	2 dissatisfied	217	32.6
	3 neither	107	16.1
	4 satisfied	207	31.1
	5 very satisfied	24	3.6
FU3 Survival	Yes	504	75.7
	No	32	4.8
	No information	130	18.5
FU6 Survival	Yes	250	57.6
	No, already at FU3	21	4.8
	No, at FU6	7	1.6
	No information	156	35.9

Note: GDS = Geriatric Depression Scale, MMSE = Mini Mental State Examination.

## Data Record

The dataset for this study is stored in an Excel and SPSS sheet for use in a multitude of statistical programs. It contains both baseline and follow-up (FU3 and FU6) information in a wide format. Data documentation is freely available to any researcher to gauge whether the data is of interest for their respective research questions^32^. However, due to the sensitive nature of the data reflecting patient responses, the dataset itself is only available by request. Data can be requested from the UK Data Service (https://reshare.ukdataservice.ac.uk/858380/) managing the controlled access; once granted, the data can be downloaded^32^.

In the dataset, missing values are indicated with blanks. Each row represents a single patient as defined by their anonymous patient code, and each column represents a variable. In the documentation and legend, detailed information on the variables and questionnaires as well as translations of the study-specific questionnaires can be obtained.

As the dataset contains sensitive information on patients that may lead to a potential identification, we performed several steps to avoid de-anonymization:

- Each patient is defined by a randomly generated patient code, no names are given
- No date of birth is given, only the numerical age at baseline is presented
- No dates of admission are given
- No individual diagnoses or medical procedures are reported, main diagnosis at admission has been grouped into 8 overarching diagnosis categories based on the ICD codes for each patient:

1. Diseases of the musculoskeletal system and connective tissue (ICD-10: M00–M99)
2. Diseases of the circulatory system (ICD-10: I00–I99)
3. Diseases of the nervous system (ICD-10: G00–G99)
4. Infectious and parasitic diseases (ICD-10: A00–B99)
5. Diseases of the genitourinary system (ICD-10: N00–N99)
6. Endocrine, nutritional and metabolic diseases (ICD-10: E00–E90)
7. Diseases of the digestive system (ICD-10: K00–K93)
8. Other

No additional qualitative information gathered during the interviews is presented

## Technical Validation

The data described in this manuscript directly reflect patients’ individual responses to questionnaires and scores on routine assessments. Therefore, depending on current functional status, experiences and expectations, responses may vary and each patients’ responses should be considered individually. Of note, the presented data stems from a vulnerable, often excluded patient population and thus cannot be compared to other healthy, younger population. To still ensure overall good data quality, we performed analyses to indicate data validity.

As a key outcome in the present study, the Appraisal for Self-Care Agency Scale (ASAS)^33,34^ measures self-management actions with 15 items. To assess its validity, we utilized the Patient-Activation Measure (PAM)^35,36^ as a measure for convergent validity. In the present dataset, ASAS and PAM correlate at r = 0.66 (p < 0.001), indicating good convergent validity. Of note, the ASAS does not vary significantly depending on the recruitment location (Table 3). Cronbachs α was accepable for ASAS at baseline (α = 0.82, CI[.81, 0.85]) and at FU (α = 0.84, CI[.80, 0.86]) as well as baseline PAM (α = 0.89, CI[.88, 0.90]).

**Table 3:** Comparison of data based on recruitment location.

Variable	HOSP1	HOSP2	HOSP3	GP	Compare
	Mean (SD)	Mean (SD)	Mean (SD)	Mean (SD)	P, η²
Age	82.3 (6.3)	84.3 (5.3)	81.7 (6.3)	79.0 (6.4)	<0.001, 0.08
Barthel Admission	37.3 (14.8)	53.5 (19.3)	44.1 (16.9)		<0.001, 0.11
Barthel Discharge	62.4 (20.5)	71.6 (17.1)	73.2 (19.4)		<0.001, 0.03
MMSE	25.1 (3.3)	25.4 (3.4)	25.1 (3.2)		.522, 0.001
GDS	3.87 (2.8)	4.0 (3.0)	4.54 (3.1)	4.4 (3.5)	.231, 0.002
ASAS	53.1 (8.0)	54.1 (9.8)	54.1 (6.5)	54.0 (9.2)	.411, 0.002
WHOQOL: Physical	53.2 (20.4)	56.2 (22.8)	46.9 (19.0)	61.5 (20.1)	<0.001, 0.05
Mental	69.9 (16.3)	70.3 (16.3)	62.4 (16.8)	67.0 (16.9)	<0.001, 0.03
Social	67.1 (19.4)	74.0 (18.4)	67.2 (14.9)	62.3 (16.7)	<0.001, 0.03
Environmental	74.9 (13.7)	74.6 (16.1)	73.8 (13.1)	69.7 (15.1)	.026, 0.01
Number of Syndromes	3.51 (1.8)	3.69 (1.8)	3.58 (1.8)	2.74 (1.9)	<0.001, 0.04
	**Count (%)**	**Count (%)**	**Count (%)**	**Count (%)**	***Chi2-Test p, V***
Health: 1 very dissatisfied	29 (19.3)	25 (10.9)	14 (14.4)	5 (3.2)	<0.01, 0.16
2 dissatisfied	42 (28.0)	92 (40.0)	31 (32.0)	52 (33.6)	
3 neither	33 (22.0)	35 (15.2)	12 (12.4)	27 (17.4)	
4 satisfied	41 (27.3)	66 (28.7)	30 (30.9)	70 (45.2)	
5 very satisfied	5 (3.3)	12 (5.2)	7 (7.2)	1 (0.7)	
Gender: Female	108 (69.7)	159 (68.5)	68 (54.9)	93 (60.0)	.02, 0.12
Male	47 (30.3)	73 (31.5)	56 (45.2)	62 (40.0)	
Education: Low	7 (4.6)	6 (2.6)	4 (3.3)	0	<0.001, 0.34
Middle	112 (73.2)	50 (21.6)	84 (67.7)	108 (71.5)	
High	34 (22.2)	176 (75.9)	36 (29.0)	46 (30.5)	
Marital: Single	16 (10.4)	12 (5.2)	6 (4.8)	10 (6.5)	.03, 0.10
Married/Partner	58 (37.7)	78 (33.6)	47 (37.9)	73 (47.7)	
Divorced/Widowed	80 (52.0)	142 (61.2)	71 (57.3)	70 (45.8)	

Note: HOSP1 and HOSP3 SelfmanGer, HOSP2 JenaGer

ASAS = Appraisal for Self-Care Agency Scale, GDS = Geriatric Depression Scale, MMSE = Mini Mental State Examination, Number of Syndromes = Number of self-reported geriatric syndromes (0-9), SRH = self-rated health, WHOQOL = WHO Quality of Life Bref questionnaire, subscales physical, mental, social and environmental

η² interpretation: 0.01- < 0.06 small, 0.06 - < 0.14 moderate, and ≤ 0.14 large

Cramer V interpretation: < 0.2 small, 0.2 – ≥ 0.6 moderate, and < 0.6 large.

Overall, 155 patients were recruited from hospital 1 and 124 from hospital 3 for the *SelfManGer* study, with additional 155 GP patients. For *JenaGer*, 232 patients were recruited from hospital 3. For all variables, differences between patients from the four locations yield only small effect sizes, indicating comparable data across recruitment locations (Table 3).

In addition to different locations, the three time-points (baseline, FU3, FU6) further warrant validation. In Table 4, we present group comparisons for baseline characteristics of responders and non-responders at FU3 and FU6. Notably, despite dropout at both FU3 and FU6, there were no substantial differences between those patients who participated at FU3 and FU6 in comparison to those who were not reached. At FU3, nonresponders differed from responders in Barthel Index at admission, GDS, and MMSE, albeit with small effect sizes. At FU6, the higher age of nonresponders was the only significant difference between the two groups, again with a small effect size.

**Table 4:** Comparison of baseline characteristics for responders and non-responders at follow up.

Time	FU3 (N = 349 vs 317)			FU6 (N = 185 vs 246)		
	Responders	Nonresp.	Comparison	Responders	Nonresp.	Comparison
**Variable**	**M (SD)**	**M (SD)**	***p*, r**	**M (SD)**	**M (SD)**	***p*, r**
Age	81.8 (6.26)	82.5 (6.30)	0.24, 0.05	79.7 (6.63)	81.9 (6.24)	0.001, 0.17
Barthel	41.8 (17.7)	43.9 (19.9)	0.01, 0.11	40.9 (14.3)	39.7 (17.7)	0.37, 0.05
GDS	3.79 (2.79)	4.57 (3.40)	0.01, 0.10	3.96 (2.80)	4.49 (3.39)	0.21, 0.061
MMSE	25.6 (3.13)	24.7 (3.49)	0.003, 0.13	25.5 (2.91)	24.8 (3.51)	0.16, 0.08
Syndromes	3.32 (1.8)	3.51 (1.89)	0.31, 0.04	3.24 (1.78)	3.28 (1.89)	0.93. 0.004
	**Count (%)**	**Count (%)**	***p*, V**	**Count (%)**	**Count (%)**	***p*, V**
Gender			0.72, 0.01			0.54, 0.03
Female	227 (65.0)	201 (63.4)		119 (64.3)	150 (61.0)	
Male	122 (35.0)	116 (36.6)		66 (35.7)	96 (39.0)	
Education			0.18, 0.07			0.51*, 0.06
Low	6 (1.72)	11 (3.47)		3 (1.62)	8 (2.25)	
Middle	181 (51.9)	173 (54.6)		129 (69.7)	173 (70.3)	
High	162 (46.4)	129 (40.7)		52 (28.1)	63 (25.6)	
Living			0.61, 0.04			0.09, 0.11
Alone	175 (50.1)	151 (47.6)		82 (44.3)	119 (48.4)	
With Family	147 (42.1)	133 (42.0)		93 (50.3)	101 (41.1)	
Nursing	27 (7.74)	31 (9.78)		10 (5.41)	24 (9.76)	
Health			0.35, 0.08			0.98*, 0.03
1	39 (11.2)	34 (10.7)		20 (10.8)	28 (11.4)	
2	106 (30.4)	111 (35.0)		53 (28.6)	72 (29.3)	
3	53 (15.2)	54 (17.0)		29 (15.7)	42 (17.1)	
4	106 (30.4)	101 (31.9)		57 (30.8)	83 (33.7)	
5	17 (4.87)	7 (2.21)		22 (11.9)	8 (3.25)	

Note:

Effect sizes for numeric variables: Pearson r

Effect sizes for categorical variables: Cramer’s V

*Fishers Exact Test performed for Health and Education at FU6.

**Fig. 2:**
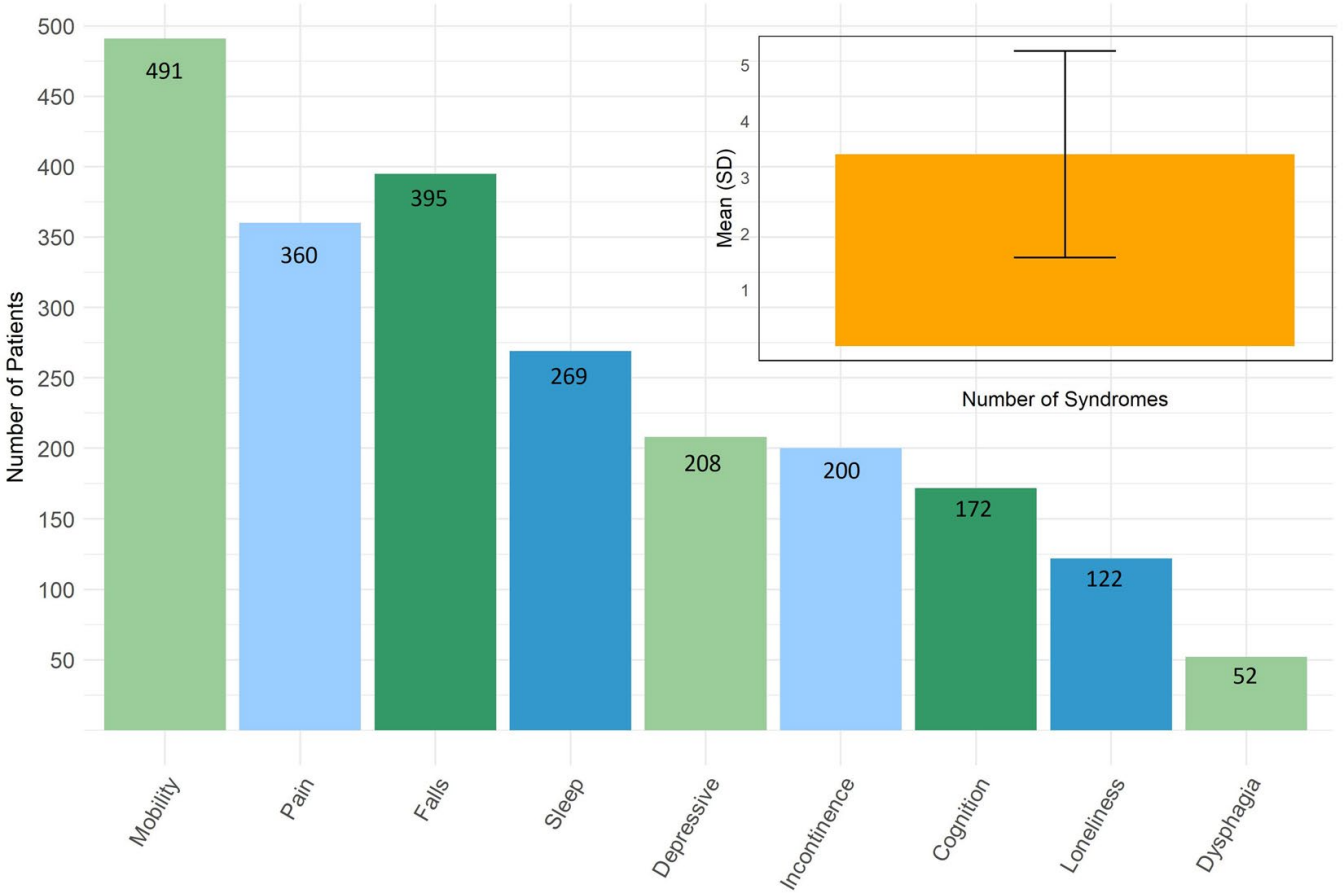
Descriptive Statistics of Geriatric Syndromes.

Regarding geriatric syndromes, previous studies varied in their included syndromes^14,18,37^, and there is no consensus on which syndromes have to be included. However, previous studies confirm our results (Fig. 2) showing that mobility-related syndromes and pain are among the most frequent. Likewise, an average of 3.4 syndromes (SD = 1.84, see Tables 1 and 3) is comparable to other studies in a similar age range^15,16,19,38^, indicating that our data on geriatric syndromes is representative of geriatric patients.

Internal Consistency of the self-reported questionnaires were high for the Generalized Self-Efficacy Scale (GSE, α = 0.95, CI[.94, 0.95]), very good for the WHO psychological subscale (α = 0.80, CI[.78, 0.83]), good for the WHO physical (α = 0.78, CI[.76, 0.81]) and environmental (α = 0.77, CI[.74, 0.80]) subscales as well as the Beck Anxiety Index (BAI, α = 0.78, CI[.74, 0.81]) and UCLA Loneliness scale (α = 0.77, CI[.74, 0.80]), and acceptable for the WHO social subscale (α = 0.67, CI[.62, 0.71]). Notably, we do not provide a measure of internal consistency for the geriatric syndromes, as each geriatric syndrome stands on its own and can be present irrespective of the other syndromes. The sum score *SyndromeNumber* is calculated as a proxy for syndrome burden, however, depending on the research question, it is recommended to consider the impact of each syndrome separately.

## Usage Notes

The presented data was collected from three hospitals and two outpatient clinics. Although an overarching study team recruited patients from all locations to reduce bias, different hospitals may be specialized in different treatments, potentially leading to differences between patients (see Table 1 and Table 3 for an overview of differences between patient groups). Thus, it is recommended to include the *Location* variable as a covariate to control for any location effects. Additionally, the locations may differ in some routine measurement instruments, e.g. in the nutritional risk screening tool and the cognition screening tool. To be able to still use the data, classification variables were created to allow for a comparison of the healthcare aspect irrespective of the utilized instrument; e.g. while the instruments to assess nutrition differ between hospitals, they all result in a classification of “at risk” vs “not at risk” which is available in addition to the item-level scores. The MiniMental State Examination (MMSE) and the Montreal Cognitive Assessment (MOCA) were both used at different locations; for comparability, all MOCA scores were transformed into MMSE scores according to the procedure described by Fasnacht *et al*.^39^. For the GP patients, the MiniCog^40^ is available as a measure of cognition. All differences between the locations are described in Table 1 as well as in detail in the *SelfManGer_Variables* document available for download with the dataset. Overall, we recommend substantiating cross-sectional with longitudinal data where possible for robust analyses and direction of effects, as well as sensitivity analyses based on location, health status, and gender. A comparison between included and excluded patients (including those with missing data) should always be reported to detect bias. Cross-sectional analyses should be theory-driven and well-discussed to arrive at meaningful conclusions.

Additionally, in some patients with severe mobility restrictions, performing the mobility assessments Tinetti and Timed Up and Go (TuG, Table 1) was not possible. This is indicated in the initial items on these assessments, clarifying whether their performance was possible or not (described in the *SelfManGer_Variables* document available with the dataset ). It is important to note that occasions in which the assessment was not performed because patients were unable to walk should not be treated as missing data, even though a final sum score does not exist. Instead, inability to perform these assessments represents the worst possible mobility state and should be included in the analyses in a way that the researcher sees fit. One solution is to categorize the variables according to recommended cut-off scores^41,42^ or by distribution-based methods, and to include one group representing inability to perform the test.

Overall, according to Little’s test of missing data, missings in routine data such as Barthel, MMSE, and GDS are missing completely at random (MCAR, p = 0.10) whereas self-reported questionnaires are not MCAR (p < 0.01). When using the dataset, researchers are invited to perform multiple imputation and compare those imputed datasets with the complete case analysis to increase the robustness of their results, and to consider sensitivity analysis depending on their research question and statistical approach. Notably, we do not provide imputed data but instead report the raw data as collected directly from the patients, in order to allow each researcher to select the best approach depending on their research question.

Certain assessments were performed multiple times as part of routine geriatric care, including the Barthel Index, Tinetti and TuG test, which are performed upon admission and again before discharge to track each patient’s progress. In the dataset, where available, these instruments are reported twice, allowing researchers to select which timepoint they wish to include in analyses.

While internal consistency as indicated by Cronbach’s α is given above, we do not report test-retest reliability of the self-reported questionnaires. This is because uncovering test-retest reliability of the utilized validates scales and its implications is out of the scope of this current manuscript. Additionally, changes in the questionnaire responses are expected and plausible in the present geriatric patient group, who suffered from an acute health event at baseline followed by either increasing or worsening health at FU timepoints. The association between each questionnaire at baseline and FU and the influential factors for changes, however, represents an important research question that must be addressed and discussed in depth using this dataset.

Lastly, for the use of follow-up data, the substantial drop-out especially at FU6 must be taken into consideration. According to group comparisons between responders and non-responders given in Table 4, differences between the two groups regarding baseline demographics and health information (subjective health, Barthel, cognition, depressiveness, geriatric syndromes) are negligible. This indicates that other variables, potentially structural hurdles such as moving into nursing homes or not wanting to be contacted due to worsening health, may play a bigger role here. This finding hints at a general challenge of recruitment in geriatric patients, suggesting that longitudinal data collection is challenging even via telephone, due to changes in health and living arrangements, as well as mortality. Although our results suggest that bias is limited as the data between responders and non-responders is comparable, in future studies on geriatric patients, it may be beneficial to shorten follow-up timeframes (e.g. after one and three months).

Overall, although this manuscript provides data on a highly vulnerable and oftentimes overlooked patient group, there is still a limitation in the representativeness of the data due to the exclusion of patients with severe dementia, delirium or physical impairment. Although methods are recommended on how to include these patients in scientific research^31^, data collection in geriatric patients is time-consuming and complex, and the present data is based primarily on questionnaires. The requirement to understand scientific questionnaires and to provide informed consent made the exclusion of severely impaired patients necessary. However, in future studies, we recommend narrowing questionnaires down to the most essential items and collecting data anonymously, or collecting at least routine data also for patients with severe cognitive or physical impairment to shed a light on all geriatric patients^31^.

## Supplementary information

Supplement_TranslatedQuestionnaires

## Data Availability

No specific code is needed to utilize the data, as all variables directly reflect the patients’ responses to each questionnaire item. However, sum scores and sub-scales are provided in the dataset, these were calculated in R Version 4.3.0^43^ according to the manuals provided by the respective authors of each measurement instrument: - WHOQOL-Bref: Scores are provided for the physical, mental, social and environmental subscale of QoL^44,45^. - Overall sum scores are provided for: ASAS^34,46^, BAI^47^, Barthel^48^, GDS^49^, GSE^50^, Lachs^51^, MMST^52^, Tinetti^42^, UCLA^53^, VOA^54^.

## Data availability

The dataset additionally contains responses to all individual items to provide researchers the opportunity to perform detailed analyses. In the SPSS dataset, correct variable scales are already provided; for R users, a corresponding script for data preparation prior to analyses is uploaded along with the dataset from Prell, T., Schönenberg, A., Heimrich, K.G. (2026). *Self-Management of Geriatric Syndromes–Longitudinal Data on Medical and Psychosocial Factors in Older Patients, 2023-2024*. [Data Collection]. Colchester, Essex: UK Data Service. https://reshare.ukdataservice.ac.uk/858380/.
