# Supplementary material for: Self-Management of Geriatric Syndromes–longitudinal data on medical and psychosocial factors in older patients
Source: Sci Data. 2026 May 28;13:794. doi: 10.1038/s41597-026-07405-x (PMC13219512; doi:10.1038/s41597-026-07405-x)
Supplement: Supplementary file 1 — Supplement_TranslatedQuestionnaires [file 41597_2026_7405_MOESM1_ESM.pdf]

Baseline

Age	______ Years
Gender	1 Feale 2 Male
Marital State	0 Single 1 Married<br>2 Widowed / Divorced, Separated
Living Situation	0 Alone 1 With partner/in Family<br>2 Other: _________________________
Education (according to the German education system)<br><br>Classified into:<br>Low Less than 10 years<br>Middle 10-12 years<br>High More than 12 years	None (less than 8 years)<br>8 years<br>10 years<br>12 years<br>More than 12 years (university degree)
Who prepares your medication?<br><br>(SelfManGer only)	0 You yourself 1 Partner 5 Other<br>4 Pharmacist 2 Family Member
Which of the following aids do you use?	Glasses Hearing Aids Voice computer<br>Walking Stick Crutches Forearm supports<br>Wheeled Walker Wheelchair Other:
How often do you see your GP per quarter?	
Which therapies do you use regularly?	Physiotherapy rehabilitation exercises Other:<br>Occupational Therapy speech therapy
Which care level do you have?	none 1 2 3 4 5
Do you use a nursing service?	1 Yes 0 No 2 No, but my relatives help
Which technological devices do you own?	Smartphone Landline<br>Laptop/Computer Tablet Other: _______________
How often do you use those devices?	0 Never 1 Monthly<br>2 Weekly 3 Daily
What do you use those devices for?	Communication<br>Information research<br>News<br>Entertainment (Movies, Music, Books)<br>Work<br>other hobbies<br>other: ___________________________________

 Date: ____________ Code: ____________

1. Which of the following challenges do you experience?
   (You can choose multiple)

☐ reduced mobility (e.g. when walking or climbing stairs)
☐ gait problems and falls
☐ cognitive restrictions, e.g. problems with memory
☐ feeling blue/sadness
☐ loneliness
☐ pain
☐ incontinence (urinal)
☐ sleep problems
☐ problems with swallowing

2. Which problem is the most restricting to you in your daily life?
   (Please only select one!)

☐ reduced mobility (e.g. when walking or climbing stairs)
☐ gait problems and falls
☐ cognitive restrictions, e.g. problems with memory
☐ feeling blue/sadness
☐ loneliness
☐ pain
☐ incontinence (urinal)
☐ sleep problems
☐ problems with swallowing

3. How restricted are you in your daily life due to the problem chosen at question 2?

0 ────────────────────────→ 100

50

0 = no restriction, I can carry out my daily activities without problems

100 = fully restricted, a normal daily life is not possible

4. Would you attribute the problem chosen at question 2 rather to age or to an illness?

0 ────────────────────────→ 100

50

0 = Illness, age has nothing to do with it

100 = Age, illness has nothing to do with it

 Date: ____________ Code: ____________

5. How worried are you that the problem chosen at question 2 will worsen?

0 ——————————————————————→ 100

50

0 = I am sure the problem will not worsen further

100 = the problem will definitely worsen

6. How confident are you that the problem chosen at question 2 will improve?

0 ——————————————————————→ 100

50

0 = I assume that the problem cannot improve

100 = the problem will definitely improve

# QUESTIONNAIRE DATA; SEE BELOW

Follow Up

Conversation partner	☐ patient ☐ Relative: ___________________<br>☐ other: _________________
Conversation possible?	☐ Yes ☐ No, reason: ☐ Nursing Home ☐ Mortality<br>☐ Health ☐ Relatives give no information<br>☐ no contact information ☐ not reached ☐ other:
Survival?	☐ Yes ☐ No
How are you feeling compared to the last time we spoke?	☐ Better ☐ Same ☐ Worse
Have you been in contact with a medical professional since we last spoke?<br><br>(JenaGer: only hospital)	☐ No<br>☐ Yes<br>☐ GP<br>☐ Specialist: ______________________________<br>☐ **Hospital stay**
Did you receive new diagnoses since we last spoke?	☐ No<br>☐ Yes
Were any changes made to your medication since we last spoke?	☐ No<br>☐ Yes
Do you partake in non-medical treatments since discharge from the geriatric ward?	☐ No<br>☐ Physiotherapy ☐ Rehabilitation classes ☐ Occupational therapy ☐ speech therapy ☐ other: ____________________
Do you newly make use of a nursing service since discharge?/since we last spoke?	☐ No/already before hospital stay<br>☐ No, but my relatives take care of me<br>☐ Yes<br>☐ I am in a nursing home
Has your care level changed since discharge?/since we last spoke?	☐0 No ☐1 Yes, new care level:<br>☐ 1 ☐ 2 ☐ 3 ☐ 4 ☐ 5
Has your mobility changed since discharge/since we last spoke?	☐ Yes, better ☐ No, same<br>☐ Yes, worse, reason: __________________________<br><br>Did you fall? ☐ No ☐ Yes
Do you have pain?	☐ No ☐ Yes, a little ☐ Yes, strongly
Do you feel like your concentration (attention, memory) has changed since discharge/since we last spoke?	☐ Yes, better ☐ No, same<br>☐ Yes, worse
How often did you fall since being discharged/since we last spoke?	____ times

REPEAT QUESTIONS ON GERIATRIC SYNDROMES FROM BASELINE

ADD
Since discharge, hast he selected syndrome… ☐ stayed the same ☐ improved ☐ gotten worse

QUESTIONNAIRE DATA

AT FU: GDS, MoCA word fluency and delayed recall, Barthel,
ASAS, WHOQOL-BREF, Views on Ageing, Beliefs about Medication Questionnaire (BMQ)

Geriatric Assessment

- Geriatric depression scale, *GDS* (Gauggel & Birkner, 1999; Yesavage et al., 1982),
- *Barthel* Index (Mahoney & Barthel, 1965),
- Geriatric screening according to *Lachs (Lachs et al., 1990)*
- Cognition according to the Montreal Cognitive Assessment, *MoCA* or the Mini Mental State Examination (MMSE) (Folstein et al., 1975; Nasreddine et al., 2005). Of note, for reasons of comparability, the MoCA was transformed to match the MMSE according to the conversion described in Fasnacht et al. (2023)
- Tinetti Test (Tinetti, 1986)
- Timed Up and Go Test (Podsiadlo & Richardson, 1991)
- Handgrip Strength measured with JAMAR Dynamometer in KG
- Nutritional Risk Screening (NRS) (Reber et al., 2019) or Mini Nutritional Assessment Short Form (MNA-SF) (Vellas et al., 1999)

Study-specific questionnaires

- Appraisal for Self-Care Agency Scale (ASAS)(Schönenberg et al., 2022; Sousa et al., 2010)
- Beck Anxiety Index (BAI) short (Beck et al., 1988)
- Beliefs about Medication Questionnaire (BMQ) (Horne et al., 1999)
- Generalized Self-Efficacy Scale (GSE) (Schwarzer & Jerusalem, 1995)
- Quality of Life (WHOQOL-Bref)(WHO, 2004, 2020)
- UCLA-Loneliness Scale- Short Form (Hughes et al., 2004; Russell, 1996)
- Views on Ageing (Engstler et al., 2019; Klaus et al., 2017)

References

Beck, A. T., Epstein, N., Brown, G., & Steer, R. A. (1988). An inventory for measuring clinical anxiety: Psychometric properties. *Journal of Consulting and Clinical Psychology*, *56*(6), 893-897. https://doi.org/10.1037/0022-006X.56.6.893

Engstler, H., Alcantara, A. L., Luitjens, M., & Klaus, D. (2019). *German Ageing Survey, Deutscher Alterssurvey (DEAS): Documentation of instruments and variables 1996 – 2017*. Deutsches Zentrum für Altersfragen. https://doi.org/10.5156/DEAS.1996-2017.D.002

Fasnacht, J. S., Wueest, A. S., Berres, M., Thomann, A. E., Krumm, S., Gutbrod, K., Steiner, L. A., Goettel, N., & Monsch, A. U. (2023). Conversion between the Montreal Cognitive Assessment and the Mini-Mental Status Examination. *J Am Geriatr Soc*, *71*(3), 869-879. https://doi.org/https://doi.org/10.1111/jgs.18124

Folstein, M. F., Folstein, S. E., & McHugh, P. R. (1975). "Mini-mental state". A practical method for grading the cognitive state of patients for the clinician. *J Psychiatr Res*, *12*(3), 189-198. https://doi.org/10.1016/0022-3956(75)90026-6

Gauggel, S., & Birkner, B. (1999). Validität und Reliabilität einer deutschen Version der Geriatrischen Depressionsskala (GDS). [Validity and reliability of a German version of the Geriatric Depression Scale (GDS).]. *Zeitschrift für Klinische Psychologie*, *28*, 18-27. https://doi.org/10.1026/0084-5345.28.1.18

Horne, R., Weinman, J., & Hankins, M. (1999). The beliefs about medicines questionnaire: The development and evaluation of a new method for assessing the cognitive representation of medication. *Psychology & Health*, *14*(1), 1-24. https://doi.org/10.1080/08870449908407311

Hughes, M. E., Waite, L. J., Hawkley, L. C., & Cacioppo, J. T. (2004). A Short Scale for Measuring Loneliness in Large Surveys: Results From Two Population-Based Studies. *Res Aging*, *26*(6), 655-672. https://doi.org/10.1177/0164027504268574

Klaus, D., Engstler, H., Mahne, K., Wolff, J. K., Simonson, J., Wurm, S., & Tesch-Römer, C. (2017). Cohort Profile: The German Ageing Survey (DEAS). *Int J Epidemiol*, *46*(4), 1105-1105g. https://doi.org/10.1093/ije/dyw326

Lachs, M. S., Feinstein, A. R., Cooney, L. M., Jr., Drickamer, M. A., Marottoli, R. A., Pannill, F. C., & Tinetti, M. E. (1990). A simple procedure for general screening for functional disability in elderly patients. *Ann Intern Med*, *112*(9), 699-706. https://doi.org/10.7326/0003-4819-112-9-699

Mahoney, F. I., & Barthel, D. W. (1965). Functional Evaluation: the Barthel Index. *Md State Med J*, *14*, 61-65.

Nasreddine, Z. S., Phillips, N. A., Bédirian, V., Charbonneau, S., Whitehead, V., Collin, I., Cummings, J. L., & Chertkow, H. (2005). The Montreal Cognitive Assessment, MoCA: a brief screening tool for mild cognitive impairment. *J Am Geriatr Soc*, *53*(4), 695-699. https://doi.org/10.1111/j.1532-5415.2005.53221.x

Podsiadlo, D., & Richardson, S. (1991). The timed "Up & Go": a test of basic functional mobility for frail elderly persons. *J Am Geriatr Soc*, *39*(2), 142-148. https://doi.org/10.1111/j.1532-5415.1991.tb01616.x

Reber, E., Gomes, F., Vasiloglou, M. F., Schuetz, P., & Stanga, Z. (2019). Nutritional Risk Screening and Assessment. *J Clin Med*, *8*(7). https://doi.org/10.3390/jcm8071065

Russell, D. W. (1996). UCLA Loneliness Scale (Version 3): reliability, validity, and factor structure. *J Pers Assess*, *66*(1), 20-40. https://doi.org/10.1207/s15327752jpa6601_2

Schönenberg, A., Teschner, U., Prell, T., & Mühlhammer, H. M. (2022). Validation and Psychometric Analysis of the German Translation of the Appraisal of Self-Care Agency Scale-Revised. *Healthcare (Basel)*, *10*(9). https://doi.org/10.3390/healthcare10091785

Schwarzer, R., & Jerusalem, M. (1995). Generalized Self-Efficacy scale. In S. Weinman, S. Wright, & M. Johnston (Eds.), *Measures in health psychology: A user's portfolio. Causal and control beliefs* (pp. 35-37). NFER-NELSON.

Sousa, V. D., Zauszniewski, J. A., Bergquist-Beringer, S., Musil, C. M., Neese, J. B., & Jaber, A. F. (2010). Reliability, validity and factor structure of the Appraisal of Self-Care Agency Scale-Revised (ASAS-R). *J Eval Clin Pract*, *16*(6), 1031-1040. https://doi.org/10.1111/j.1365-2753.2009.01242.x

Tinetti, M. E. (1986). Performance-oriented assessment of mobility problems in elderly patients. *J Am Geriatr Soc*, *34*(2), 119-126. https://doi.org/10.1111/j.1532-5415.1986.tb05480.x

Vellas, B., Guigoz, Y., Garry, P. J., Nourhashemi, F., Bennahum, D., Lauque, S., & Albarede, J. L. (1999). The Mini Nutritional Assessment (MNA) and its use in grading the nutritional state of elderly patients. *Nutrition*, *15*(2), 116-122. https://doi.org/10.1016/s0899-9007(98)00171-3

WHO. (2004). The World Health Organization's WHOQOL-BREF quality of life assessment: psychometric properties and results of the international field trial. A report from the WHOQOL group. *Quality of Life Research*, *13*(299-310).

WHO. (2020). *WHOQOL: Measuring Quality of Life*. World Health Organization. Retrieved 11.04. from https://www.who.int/toolkits/whoqol

Yesavage, J. A., Brink, T. L., Rose, T. L., Lum, O., Huang, V., Adey, M., & Leirer, V. O. (1982). Development and validation of a geriatric depression screening scale: a preliminary report. *J Psychiatr Res*, *17*(1), 37-49. https://doi.org/10.1016/0022-3956(82)90033-4
